# Interaction Effects of Season of Birth and Cytokine Genes on Schizotypal Traits in the General Population

**DOI:** 10.1155/2017/5763094

**Published:** 2017-12-31

**Authors:** Margarita V. Alfimova, Galina I. Korovaitseva, Tatyana V. Lezheiko, Vera E. Golimbet

**Affiliations:** Mental Health Research Center, Kashirskoe Shosse 34, Moscow 115522, Russia

## Abstract

Literature suggests that the effect of winter birth on vulnerability to schizophrenia might be mediated by increased expression of proinflammatory cytokines due to prenatal infection and its inadequate regulation by anti-inflammatory factors. As the response of the immune system depends on genotype, this study assessed the interaction effects of cytokine genes and season of birth (SOB) on schizotypy measured with the Schizotypal Personality Questionnaire (SPQ-74). We searched for associations of* IL1B* rs16944,* IL4* rs2243250, and* IL-1RN* VNTR polymorphisms, SOB, and their interactions with the SPQ-74 total score in a sample of 278 healthy individuals. A significant effect of the* IL4* X SOB interaction was found, *p* = 0.007 and *η*^2^ = 0.028. We confirmed this effect using an extended sample of 373 individuals. Homozygotes CC born in winter showed the highest SPQ total score and differed significantly from winter-born T allele carriers, *p* = 0.049. This difference was demonstrated for cognitive-perceptual and disorganized but not interpersonal dimensions. The findings are consistent with the hypothesis that the cytokine genes by SOB interaction can influence variability of schizotypal traits in the general population. The* IL4* T allele appeared to have a protective effect against the development of positive and disorganized schizotypal traits in winter-born individuals.

## 1. Introduction

Evidence suggests that people born during winter months have an increased risk of developing schizophrenia [1–3]. However, the definitive pathophysiological mechanisms underlying the link between season of birth (SOB) and the disorder are still not completely understood. One plausible explanation is that the increased risk of schizophrenia in individuals born in winter is caused by deleterious effects of innate immune response-related factors on brain development [2, 4, 5]. Recent findings have demonstrated that SOB may shape neonatal immune function, with winter births correlating with higher levels of most immune cell types in cord blood and airway inflammatory mediators, and this season-related immune profile seems to be an outcome of exposures from the maternal environment [6]. Accordingly, epidemiological data implicate maternal infection as a risk factor for schizophrenia, and animal models corroborate this link showing the impact of maternal immune activation on lifelong neuropathology in offspring [7, 8]. Since prenatal exposure to different types of viral or bacterial infections has similar debilitating effects in later life, it is thought that not pathogen itself but the response of the innate immune system, specifically the increased production of inflammatory cytokines, is the critical mediator in altering fetal brain development [9].

Schizophrenia is considered to be a neurodevelopment disorder resulting from complex interactions between genes and environmental exposures [10]. In this context, it is suggested that genotype may mediate prenatal or early postnatal environmental influences that activate the immune system on schizophrenia-related brain pathology [11]. However, while recent years have seen considerable progress in uncovering environmental and genetic factors predisposing to schizophrenia, only a limited number of studies have investigated gene-environment interactions (G × E). Of them, four studies have used SOB as a proxy of environmental exposures, with two studies exploring genes for the human leukocyte antigens and none analyzing cytokine genes (for reviews see [12–14]).

Investigating G × E in schizophrenia is challenging for a number of reasons [13]. It has been proposed that schizotypy promises to increase the power of such studies [15–17]. Schizotypy refers to a constellation of personality traits and perceptual experiences that resemble positive, negative, and disorganized symptoms of schizophrenia. It can be assessed using clinical interviews or self-report questionnaires. Individuals with high levels of schizotypal traits have an enhanced risk of psychosis, and the psychosis continuum hypothesis posits that the same biological factors that underpin schizotypy contribute to the development of schizophrenia. Therefore, schizotypy represents a useful construct for understanding the etiology of schizophrenia-spectrum disorders by allowing for the examination of etiological factors without disease-related confounders and enhancing a possibility of identifying protective mechanisms (for reviews see [15–19]).

Both quantitative and molecular genetic studies indicate overlap of schizotypy with schizophrenia at the genetic level supporting the continuum hypothesis [16, 17, 20]. About SOB, findings are mixed [21–24]. While the latest meta-analysis does not indicate an association between winter SOB and schizotypy in adults (*n* = 481) [25], in the largest study to date (*n* = 8114), a small, but significant, sex-independent effect of late winter and early spring births on schizotypal traits has been found [24]. To our knowledge, no gene X SOB study for schizotypal traits has been published so far.

This study aimed to assess the cytokine genes by SOB interaction effect on self-reported schizotypy measured with the Schizotypal Personality Questionnaire (SPQ-74) [26] in the general population. We focused on the proinflammatory cytokine interleukin- (IL-) 1*β* because of (1) its suggestive role in altered brain development [27] and schizophrenia etiology and pathogenesis [28, 29] and (2) findings indicating its higher concentration in infants born in winter [6]. Specifically, the* IL1B* gene promoter single nucleotide polymorphism (SNP) C-511T (rs16944) has been shown to be associated with schizophrenia in European populations, though the data are not univocal [30–33] and to affect brain structure and functions in schizophrenic patients [34–36]. Based on the direction of the* IL1B* allele effects on schizophrenia risk [30, 32, 33], we hypothesized that the variant C of* IL1B* would potentiate the development of schizotypal traits in individuals born during winter.

The cytokine hypothesis of schizophrenia posits that the imbalance between pro- and anti-inflammatory cytokine signaling in the fetal brain rather than an increase in proinflammatory cytokines concentration as such may represent a key mechanism involved in the precipitation of schizophrenia-related pathology [5]. Given this, we included two more genes for analysis, namely,* IL-1RN*, encoding interleukin-1 receptor antagonist (IL-1ra), and* IL4*, encoding interleukin-4. They were chosen due to the antagonistic relationships of their products with IL-1*β*. IL-1ra acts as an antagonist by binding competitively to the same membrane receptor as IL-1*β*. Like* IL1B*,* IL-1RN* is located within the 2q13 chromosomal region for which significant evidence of linkage to schizophrenia has been found [37]. IL-4 is an anti-inflammatory cytokine that can inhibit synthesis of IL-1*β*, attenuate IL-1*β*-induced behavioral and immunological changes, and upregulate the production of IL-1ra [38–40]. To examine the possible influence of these genes on schizotypal traits individually and in combination with* IL1B* and SOB, we genotyped polymorphisms with functional consequences: a penta-allelic polymorphic site in intron 2 (VNTR, 86 bp repeats) of the* IL-1RN* gene and rs2243250 in the* IL4* gene. Allele 2 (two repeats) of the* IL-1RN* gene has been related to lower IL-1ra production and a shorter time for endothelial cells' division compared with major allele 1 [41]. rs2243250 is a T/C SNP located in the* IL4* gene promoter; the minor allele T is associated with the increased* IL4* transcriptional activity [42]. We hypothesized that the potential SOB X* IL1B* effect on schizotypy would be less pronounced in the presence of the* IL-1RN* and/or* IL4* more active alleles.

## 2. Materials and Methods

### 2.1. Sample and Procedure

The study was approved by the Mental Health Research Center's Ethics Committee. Subjects were recruited as part of larger research on the genetics of psychiatric disorders. We selected participants from the staff of research institutes and hospitals, university students, and friends, mostly from the Moscow region (~80%). They did not receive a participation fee. Each individual was interviewed about his/her demographic characteristics, psychiatric diagnoses, substance use, or heard injury personal histories and a family history of psychoses. Individuals who were not Caucasian, did not complete a secondary school (11 years), reported a history of psychiatric or neurologic conditions, or had first-degree relatives with psychotic illness were not included in the sample. The entire research design required subjects to sign an informed consent for participation in the study, to donate blood samples for DNA extraction, and to complete a set of inventories including the SPQ-74.

A two-stage design was applied. In stage 1, a search for associations of all the three cytokine genes, SOB, and their interactions with the SPQ-74 total score was performed. In stage 2, we used an extended sample to conduct follow-up analyses of the significant gene X SOB effect (1) on the SPQ total score with age and gender taken into account and (2) on each of the three SPQ factors.

Two hundred seventy-eight individuals aged between 16 and 65 years with complete genetic, demographic, and SPQ data were included in stage 1 sample. Among them, 75 participants were born in winter (December to February). The winter SOB group did not differ from the nonwinter one on age but included fewer women (52% versus 65.5%; *χ*^2^ = 4.24, *p* = 0.039). Power analysis conducted by Quanto [43] showed that the sample had over 80% power to detect main and two-way interaction effects with *R*^2^ ≥ 0.03, assuming a dominant model, a minor allele frequency of 18% or higher, and *α*  =  0.05. The extended sample (stage 2) consisted of 373 individuals approaching 80% power to detect effects of *R*^2^ ≥ 0.02 and remaining similar in age, gender, and SOB composition to the initial sample (Table 1).

### 2.2. Assessment

The SPQ-74 is a 74-item true-false questionnaire assessing nine diagnostic criteria of schizotypal personality according to DSM-III-R. The criteria are grouped into three factors: cognitive-perceptual (ideas of reference, odd beliefs or magical thinking, unusual perceptual experiences, and suspiciousness), interpersonal (social anxiety, no close friends, constricted affect, and suspiciousness), and disorganized (odd or eccentric behavior and odd speech). These factors presumably correspond to three syndromes of schizophrenia, positive (delusions and hallucinations), negative (flattened affect, avolition, and asociality), and disorganized (thought incoherence or illogicality and bizarre behavior).

### 2.3. Genotyping

DNA was extracted from peripheral blood leukocytes with the phenol-chloroform method. Genotyping was performed using polymerase chain reaction (PCR). PCR products for* IL1B* rs16944 and* IL4* rs2243250 were analyzed using restriction fragment length polymorphism (RFLP). PCR amplifications were performed in a final volume of 15 *μ*l containing 1x PCR buffer, 2.5 mM of MgCl2, 200 *μ*M dNTPs, 10.0 pmol of oligonucleotides, 0.5 U of Taq-polymerase, and 100 ng of DNA extract. Primer sequences, PCR programs, restriction endonucleases, and PCR or digestion products are presented in Table 2. For quality control purposes, 10% of samples were genotyped twice (the results were concordant). Both SNPs conformed to Hardy-Weinberg equilibrium (HWE), *p* > 0.05. Minor allele frequencies were 0.32 and 0.18 for* IL1B* and* IL4*, respectively. Genotype frequencies were as follows. For rs16944, CC = 131, CT = 118, and TT = 29; for rs2243250, CC = 184, CT = 86, and TT = 8. For* IL-1RN*, the genotype frequencies deviated from HWE (ARLEQUIN, exact test using a Markov chain, *p* = 0.042). Yet, allele 1 was the most common (0.63), followed by the two-repeat allele (0.35), consistent with the data reported for other cohorts of European ancestry (e.g., [44]).

### 2.4. Statistical Analyses

The SPQ total score was normally distributed according to the Kolmogorov-Smirnov test. A full factorial multivariate analysis of variance (ANOVA) was conducted to analyze it. The three polymorphisms and SOB (winter versus nonwinter) were used as between-subjects factors. Also, all possible two-, three-, and four-way interactions between the factors were included in the model. Genotypes were grouped as follows: at least one minor allele of any SNP versus homozygosity for its major allele; the presence of the* IL-1RN* allele 2 versus its absence. Partial eta squared (*η*^2^) was calculated to investigate effect sizes.

SPQ factor scores did not follow a normal distribution, and log-transformation failed to normalize the data. Given this, they were analyzed with the nonparametric Mann–Whitney test. Bonferroni correction was applied to *p* values during post hoc comparisons of the SPQ total score and when analyzing factor scores. In the latter case, *p*_corr_ = *p∗*3. The significance level for *p*/*p*_corr_ values was set at 0.05, two-tailed. All analyses were performed with Statistica 12 for Windows.

## 3. Results

### 3.1. Stage 1

A factorial ANCOVA with SOB and gender as between-subjects factors and age as a covariate did not reveal any significant effect of these demographic variables on the SPQ total score. The winter SOB group did not differ from the nonwinter one on the SPQ factor scores.

The full factorial ANOVA with all three polymorphisms, SOB, and their interactions as between-subjects factors yielded a significant effect of the* IL4* X SOB interaction on the SPQ total score, *F*(1,262) = 7.45, *p* = 0.007, and *η*^2^ = 0.028. T allele carriers born in winter had the lowest mean score and those who were also born in winter but did not have this allele showed the largest score (Table 3). The post hoc LSD analysis revealed nominally significant differences between carriers and noncarriers of the T allele among individuals born in winter (*p* = 0.023) and between carriers of the T allele with winter and nonwinter births (*p* = 0.045). None of these differences survived Bonferroni correction. None of the other effects in the full factorial ANOVA was significant.

### 3.2. Stage 2

A factorial ANCOVA with* IL4* genotype, SOB, and gender as between-subjects factors and age as a covariate confirmed the influence of the* IL4* X SOB interaction on the SPQ total score, *F*(1,364) = 4.88, *p* = 0.028, though the *η*^2^-value was reduced to 0.013. Homozygotes CC born in winter showed the highest SPQ total score and differed significantly from winter-born T allele carriers (*p*_corr_ = 0.049). The latter had the lowest mean score among all* IL4* X SOB groups (Table 4). In addition, the genotype main effect was significant, *F*(1,262) = 6.00, *p* = 0.015, and *η*^2^ = 0.016. Homozygotes CC had higher SPQ total scores than T allele carriers. None of the other effects in the model was significant.

Given these results, we carried out the comparisons between T allele carriers and noncarriers on the three SPQ factors in the winter SOB group. Homozygotes CC showed significantly higher scores of the cognitive-perceptual (*U* = 488.5, *p*_corr_ = 0.009) and disorganized (*U* = 508.5, *p*_corr_ = 0.017) factors than T allele carriers (Table 4). Analyses of the* IL4* effect on the SPQ factors in the whole sample and individuals born during spring-autumn did not reveal genotype-related significant differences.

## 4. Discussion

Existing literature suggests that the effect of winter birth on schizophrenia risk might be mediated by increased expression of the proinflammatory cytokines, in particular, IL-1*β*, due to prenatal infection and its inadequate regulation by anti-inflammatory factors. Therefore, we hypothesized that winter birth, combined with the presence of the schizophrenia-risk* IL1B* genotype CC, would be associated with an increase in the severity of schizotypal traits and that this effect could be modulated by genetic polymorphisms of the anti-inflammatory cytokines IL-1ra and IL-4. None of these hypotheses was confirmed. Instead, we found a significant* IL4* X SOB interaction effect on schizotypy. Among individuals born in winter, homozygotes for the common allele C had elevated SPQ scores and carriers of the more active T allele demonstrated a lower level of schizotypy. At the same time, individuals born in spring-autumn showed average levels of schizotypal traits regardless of* IL4* genotype. Based on this pattern of results, we speculate that potentially detrimental consequences of winter birth can be attenuated by increased activity of the anti-inflammatory cytokine IL-4. This hypothesis is in line with evidence of neuroprotective [45, 46] and beneficial cognitive effects of IL-4 in animal models [47, 48] and children [49]. Mechanisms underlying these effects are currently under investigation [39, 45, 50–52]. In particular, the ability of IL-4 to inhibit IL-1*β*-induced central glial activation and neurotransmitter alterations has recently been shown [39]. It can be suggested that despite the absence of the association of* IL1B* with SPQ scores in the present study, it is still possible that IL-4 exerts its protective effect against schizotypy through blocking the negative influences of IL-1*β* on normal brain development. The lack of an association between* IL1B* and schizotypy could be explained by the fact that regulation of the inflammatory response may be more important for brain development than the inflammatory reaction itself.

Factors underlying the SOB influence on schizophrenia vulnerability are not known. Apart from maternal infection, they may include perinatal photoperiod and production of vitamin D, which depends on exposure to sunlight [2, 53]. Growing evidence suggests immunomodulating effects of vitamin D [54, 55], including vitamin D ability to upregulate expression of IL-4 [54, 56–58]. As such, it can be speculated that wintertime vitamin D insufficiency can exacerbate the unfavorable effects of the less effective genotype CC on brain development. It should be noted, however, that both low and high concentrations of neonatal vitamin D are associated with increased risk of schizophrenia [59]. Further, the photoperiod might affect schizophrenia risk regardless of vitamin D, for instance, through the seasonal and circadian fluctuation of the melatonin level. Melatonin is involved in T-cell biology and maintenance of normal pregnancy [60, 61]. Treatment with melatonin has been shown to reduce levels of a number of cytokines including IL-4 in a murine model of allergic asthma [62] and to reverse ketamine-induced schizophrenia-like behavioral alterations and increments in hippocampal IL-4 in mice [63]. Finally, the actual causal event making individuals born in winter vulnerable to schizophrenia could have occurred anytime during gestation. This, in turn, suggests that factors, which are more widely dispersed in the warmer months, for example, environmental toxins [2], can be responsible for the* IL4* association with schizotypy in individuals with winter SOB.

We found that, in individuals born in winter,* IL4* was associated with positive (cognitive-perceptual) and disorganized but not negative (interpersonal) schizotypy dimensions. These results are consistent with the notion that positive and negative schizotypal features may have at least partially specific genetic and environmental underpinning [64–66]. Interestingly, Venables [67] showed that women's exposure to influenza in the 2nd trimester of pregnancy was associated with an elevation of positive schizotypy scores, whereas exposure to low environmental temperatures was associated with an elevation of anhedonia scores in their offspring. Therefore, dimensional perspective seems promising in addressing the molecular mechanisms underlying G × E in schizotypy.

The results must be interpreted in light of the limitations of our sample. First, although our sample size was moderate in comparison to other G × E studies, a larger sample size would increase power to detect three- and four-way interactions. Second, due to the very low frequency of the* IL4* TT genotype we combined individuals with TT and TC genotypes. This makes it impossible to distinguish the effects of the T allele from the effects of heterozygosity, which itself can have a beneficial effect known as a heterozygous advantage. Third, convenience sampling used in the present study is not capable of recruiting statistically representative samples of the entire population. This might limit the generalizability of the findings. However, the composition of our samples showed no bias with respect to* IL4* genotypes and SOB. Finally, other cytokine genes need to be considered in future studies. Because many anti-inflammatory molecules share similar signal transduction pathways, it may be expected that in addition to IL-4 the enhanced expression of other anti-inflammatory cytokines may also weaken or block the negative influences of winter SOB on brain development, reducing the severity of schizotypal traits in later life.

## 5. Conclusions

This study was the first to investigate the cytokine genes X SOB effects on schizotypal traits. We explored polymorphisms of* IL1B*,* IL-1RN*, and* IL4* genes and found the* IL4* X SOB influence on self-reported schizotypy. The* IL4* T allele appeared to have a protective effect against the development of positive and disorganized schizotypal traits in individuals born during winter months. We were able to replicate the* IL4* X SOB interaction effect using an extended sample, a methodological strength that places greater confidence in the findings. Further studies of the molecular mechanisms underlying the* IL4* X SOB interaction might suggest novel immunomodulatory strategies for prevention of schizophrenia. Nevertheless, replication on independent samples is desirable and more research should be conducted considering cytokine networks to find abnormalities in specific immune pathways involved in the development of schizotypal traits.

## Figures and Tables

**Table 1:** Sociodemographic characteristics of participants.

Variables	Stage 1	Stage 2
*N*	278	373

Age (M, *SD*, years)	35.1 *12.8*	33.4 *12.2*
Sex (women, *N*, %)	172 (62%)	223 (60%)
Season of birth (*N*, %)		
Winter	75 (27%)	92 (25%)
Spring	67 (24%)	94 (25%)
Summer	68 (24.5%)	88 (24%)
Autumn	68 (24.5%)	99 (26%)
SPQ total score	17.72 *10.85*	17.25 *11.13*
Cognitive-perceptual factor	7.51 *5.65*	7.13 *5.62*
Interpersonal factor	8.44 *5.77*	8.13 *5.82*
Disorganized factor	3.88 *3.13*	3.98 *3.30*

*Note*. SPQ: Schizotypal Personality Questionnaire.

**Table 2:** Genotyping conditions for the *IL1B* rs16944, *IL4* rs2243250, and *IL-1RN* VNTR polymorphisms.

Polymorphism	Primer	PCR reaction mixture and program	Restriction endonuclease	PCR or digestion products
*IL1B* rs16944	Forward 5′TGGCATTGATCTGGTTCATC3′ reverse 5′GTTTAGGAATCTTCCCACTT3′	94°C/2 min 30 cycles: 94°C/20 s 50°C/20 s 72°C/20 s 72°C/4 min	Ama87I (SybEnzym, Russia)	Digested C allele, 190 + 115 bp, nondigested T allele, 305 bp

*IL4* rs2243250	Forward 5′ACTAGGCCTCACCTGATACG3′ reverse 5′GTTGTAATGCAGTCCTCCTG3′	94°C/2 min 30 cycles: 94°C/30 s 50°C/30 s 72°C/30 s 72°C/4 min	BslFI (SybEnzym, Russia)	Digested C allele, 177 + 18 bp, nondigested T allele, 195 bp

*IL-1RN* VNTR	Forward 5′-CTCAGCAACACTCCTAT-3′ reverse 5′-TCCTGGTCTGCAGGTAA3′	94°C/2 min 30 cycles: 94°C/20 s 50°C/20 s 72°C/20 s 72°C/4 min	-	Allele 1, 412 bp, allele 2, 240 bp, allele 3, 498 bp, allele 4, 326 bp, allele 5, 584 bp

**Table 3:** Means and *SD* of the SPQ total score by SOB and genotypes.

Polymorphism	Winter SOB		Nonwinter SOB	
*IL1B* rs16944	CC homozygotes *n* = 33 16.64 *11.24*	T allele carriers *n* = 42 18.69 *8.95*	CC homozygotes *n* = 98 17.76 *11.64*	T allele carriers *n* = 105 17.64 *10.77*

*IL4* rs2243250^1^	CC homozygotes *n* = 55 19.53 *9.73*	T allele carriers *n* = 20 13.00 *9.35*	CC homozygotes *n* = 129 17.20 *11.26*	T allele carriers *n* = 74 18.54 *11.03*

*IL-1RN *VNTR	Allele 2 noncarriers *n* = 28 16.79 *10.48*	Allele 2 carriers *n* = 47 18.38 *9.77*	Allele 2 noncarriers *n* = 92 17.64 *11.41*	Allele 2 carriers *n* = 111 17.74 *11.02*

*Note*. SPQ: Schizotypal Personality Questionnaire; SOB: season of birth. ^1^The effect of the *IL4* X SOB interaction on the SPQ total score is significant, *p* < 0.01.

**Table 4:** Means and *SD* of the SPQ total and factor scores by SOB and *IL4* genotype in the extended sample.

SPQ scores	Winter SOB		Nonwinter SOB	
	CC homozygotes *n* = 68	T allele carriers *n* = 24	CC homozygotes *n* = 173	T allele carriers *n* = 108
SPQ total score	20.59 *10.36*	13.58 *10.04*^*∗*^	16.75 *11.39*	16.78 *11.09*
Cognitive-perceptual factor	8.37 *4.66*	5.42 *4.29*^*∗∗*^	6.71 *5.54*	7.41 *6.40*
Interpersonal factor	9.28 *5.95*	7.04 *5.77*	8.17 *5.92*	7.57 *5.53*
Disorganized factor	5.06 *3.79*	2.83 *2.37*^*∗*^	3.83 *3.33*	3.81 *2.94*

*Note*. SPQ: Schizotypal Personality Questionnaire; SOB: season of birth. Differences between carriers of CC genotype and T allele are significant in the winter SOB group: ^*∗*^Bonferroni adjusted *p* < 0.05. ^*∗∗*^Bonferroni adjusted *p* < 0.01.
